# Effect of community based health education on knowledge and attitude towards iron and folic acid supplementation among pregnant women in Kiambu County, Kenya: A quasi experimental study

**DOI:** 10.1371/journal.pone.0224361

**Published:** 2019-11-25

**Authors:** Mary Kamau, Waithira Mirie, Samuel Kimani, Isaac Mugoya

**Affiliations:** 1 School of Nursing Sciences, University of Nairobi, Nairobi, Kenya; 2 John Snow Inc., Nairobi, Kenya; University of Dhaka, BANGLADESH

## Abstract

**Introduction:**

Iron and Folic Acid Supplementation (IFAS) services are currently provided free of charge to pregnant women in Kenya during antenatal care (ANC) but compliance remains low. Poor awareness is an important factor contributing to low utilization of IFAS. Inadequate counselling is one of the key factors associated with poor awareness on IFAS. Community based health education is a promising diversification strategy for IFAS health education to curb this problem.

**Objectives:**

To determine effect of community based IFAS health education, utilizing CHVs, on IFAS knowledge, levels of counselling on various IFAS topics and attitude towards IFAS among pregnant women in Kiambu County.

**Methodology:**

A Pretest-Posttest Quasi-Experimental study design, consisting of intervention and control group, was applied among 340 pregnant women 18–49 years, in five health facilities, selected using two stage sampling in Lari Sub-County, Kiambu County, Kenya. Community health volunteers provided IFAS health education with weekly supplements and follow-ups to pregnant women in intervention group, while control group received the same from health care providers. Baseline and endline data were collected during ANC and compared. Quantitative data was analyzed using STATA version 14. Analysis of effect of intervention was done using Difference-In-Difference approach.

**Results:**

There was an effect difference in maternal IFAS knowledge of 13%, with intervention group levels increasing most by 35 percentage points. The odds of being knowledgeable were 3 times more at endline than baseline. There was significant (p<0.001) change in proportion with positive attitude towards IFAS: the odds of having positive attitude at endline was 9 times that of baseline (OR = 9.2:95%CI 3.1, 27.2).

**Conclusion:**

Implementation of community based health education improved maternal knowledge, positive attitude and proportion of pregnant women counselled on IFAS, better improvement being recorded in intervention group. Hence, there is need to integrate community based approach with antenatal IFAS distribution to improve supplementation.

## Introduction

Iron and Folic Acid Supplementation (IFAS) is a key intervention for prevention and control of anaemia during pregnancy [1]. This is due to increased demand for nutrients during pregnancy, especially iron and folic acid. This increase in demand is not usually met by regular diet particularly in the developing nations due to either inadequacy or reduced bioavailability of these micronutrients. There is therefore need for supplementation to prevent anaemia in pregnancy.

Kenya has implemented oral IFAS since 2010 [2] and has put efforts to improve its uptake including adoption of a combined Iron and Folic Acid (IFA) tablet in 2012. The combined tablet has less iron than the separate iron tablet previously used and was associated with most side effects. The combined IFA tablet also reduced the number of required daily tablets intake which was expected to improve compliance. Other efforts to improve IFAS uptake include (a) provision of IFAS tablets free of charge to pregnant women attending prenatal clinics in all public health facilities (b) development of IFAS policy guidelines and (c) development of Information, Education and Communication (IEC) materials for health education. Despite these efforts, the guidelines and IEC materials are not always available at the peripheral health facilities where they are needed [3]. Consequently, there is inadequate knowledge among health workers and therefore inadequate health education to pregnant women. This calls for diversification of strategies to ensure quality health education is provided on IFAS.

Various factors influence the use or non-use of antenatal IFAS services including individual socio-cultural circumstances, knowledge, attitude and practices [4–6]. Attitude is greatly influenced by the information one has and vice versa. Accordingly, the knowledge and attitude affects practice [7]. Factors that shape attitudes towards IFAS include awareness, religion, family, relatives, and friends among others. Knowledge, attitude and practice go hand in hand and subsequently influence each other. This study determined the effect of community based health education on both knowledge and attitude towards IFAS.

Poor awareness about iron/folate deficiency and anaemia is a major factor leading to low levels of IFAS knowledge and consequently low compliance with IFAS during pregnancy in developing countries [8–10]. Poor awareness on IFAS has been significantly associated with low or no use of ANC services [11]. In Kenya, many pregnant women start attending antenatal care clinics late [12]. Since ANC attendance is the main opportunity for IFAS supplementation as well as health education, most women do not get the full benefit [13]. In addition, limited knowledge and/or lack of information on IFAS has been reported among Kenyan health workers [3, 12]. Furthermore, most health care providers are overworked. Heavy workload for health care providers means that they do not have much time to counsel clients. This, coupled with limited knowledge and poor quality counselling, lead to low IFAS knowledge, awareness and eventually utilization among pregnant women [14].

Inadequate counselling is associated with poor awareness and knowledge on IFAS and therefore low IFAS utilization prenatally [11, 15]. One of the causes of inadequate counselling is poor counselling skills among health care providers and therefore an important barrier to effective supplementation. Provision of adequate information and education on health benefits of IFAS in pregnancy is important because it is a prerequisite for better IFAS utilization [15, 16] with longer duration [17], higher adherence [18] and eventual adequate supplementation [19]. A study in Thika, Kenya, showed that only about half of the respondents (45 to 58%) were provided with any information about IFAS [17, 18] while in Iran, respondents knowledge on anaemia was low [17]. Accompanying supplementation with communication was shown to improve IFAS compliance leading to decreased anaemia among adolescent girls in Tanzania [20]. Therefore, proper counselling contributes to reduction in anaemia in pregnancy by improving adherence to daily IFAS.

One of the promising strategies for diversification of health education on IFAS to minimize the problem of inadequate counselling and poor awareness is use of community based health education using community health workers. This is as a result of the understanding that good network of health facilities with well-intentioned health programmes may not necessarily lead to success of the health interventions. Success of community based interventions is determined by community involvement and ownership. This calls for clear understanding of processes and structures in a community before an intervention. Community health workers act as a bridge to link communities and health care systems. They facilitate access to health education and other health services by clients in their local contexts. With proper training, community health workers are able to provide culturally appropriate and acceptable health education and nutrition information, which is effective for the population they are serving and sensitive to clients’ experiences and values.

Use of community based channels to provide health education on anaemia and IFAS has proved successful in various countries including Cambodia, Vietnam and India where improvement in awareness and knowledge of anaemia and IFAS was observed [10]. Several community based channels have been recommended for IFAS intervention [21], most of which were used in this study, as outlined in the methodology. Clients tend to learn better when in their usual environment and this study relied on individualized visits thus the use of weekly home visits in this study in order to broaden communication efforts to improve understanding of the importance of IFAS. This helps to address misconceptions and fears about IFAS.

In Kenya, community based health education on IFAS, using regular home visits by Community Health Volunteers (CHVs) has not been practiced. Community health workers are closer to the clients and have more time with the client, to provide detailed health education and counselling. However, the effectiveness of utilizing CHVs to provide community based IFAS health education has not been determined in Kenya. The aim of this study therefore was to determine the effect of community based health education on: IFAS knowledge, levels of counselling on various IFAS aspects and attitude towards IFAS; and to determine the source of IFAS information among pregnant women, before and after community based health education.

## Materials and methods

### 2.1 Study design

**Fig 1:**
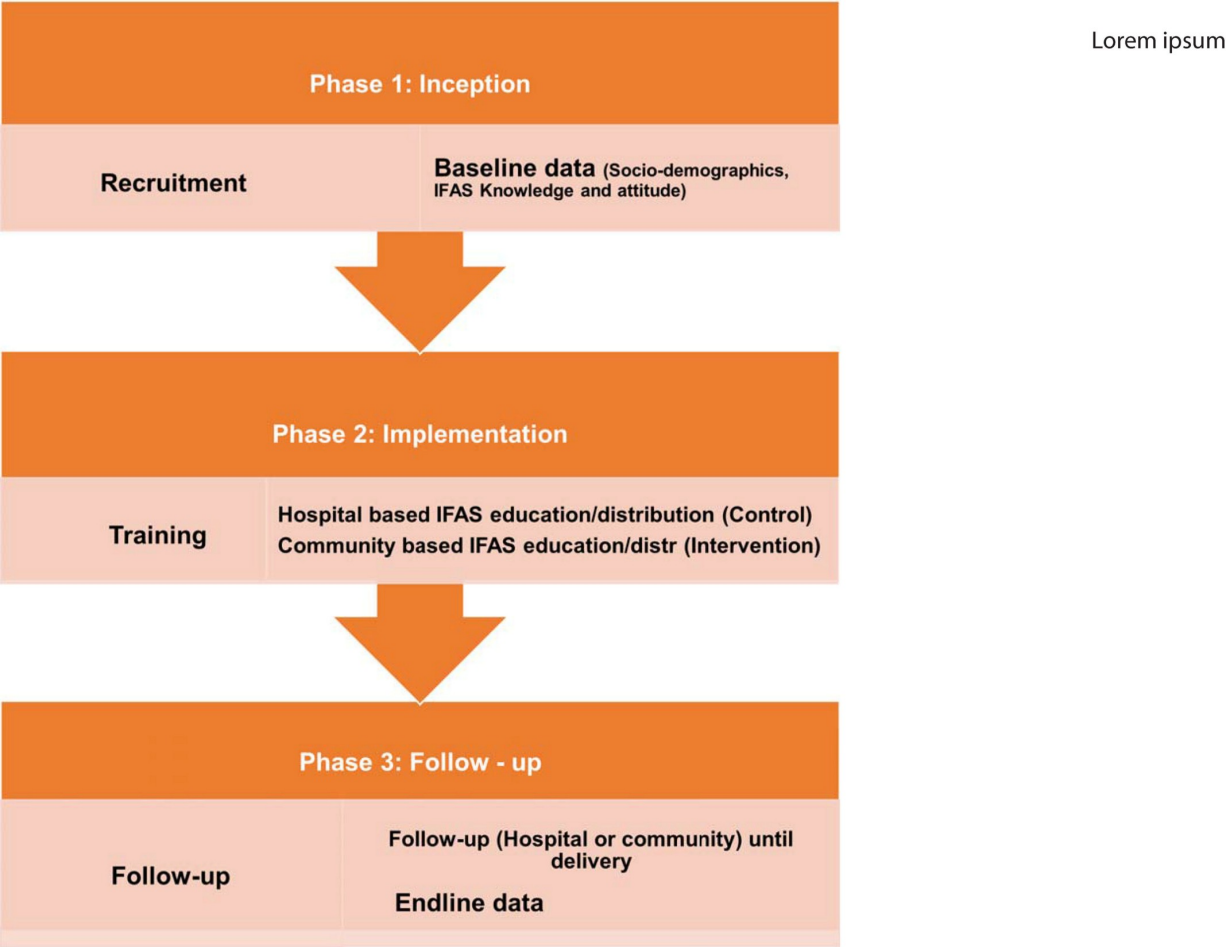
Study phases. This refers to the three stages that were followed in the implementation of this study.

This was a pretest-posttest quasi-experimental study design with a control group, used to implement a Community Based Approach (CBA) of IFAS health education in three phases namely inception, implementation and follow-up phases (Fig 1). The first phase was inception phase where study respondents were identified and recruited then baseline data was collected: on socio-demographic characteristics, IFAS knowledge, source of IFAS information and levels of counselling on various IFAS aspects, and attitude towards IFAS, among pregnant women.

The second phase was implementation phase which involved training then provision of IFAS health education together with the supplements. For the control group, health care providers, mostly nurses, were taken through a refresher training on IFAS programme. They were then provided with information, communication and education materials on IFAS developed by the Ministry of Health including: health workers training guide; national policy guideline on combined Iron and Folic Acid (IFA) supplementation for pregnant mothers in Kenya; mothers leaflets, in both English and Swahili; dialogue guide for health care providers and assorted posters. The nurses continued providing IFAS health education as well as supplements during antenatal care, as is routinely practiced. For the intervention group, Community Health Volunteers (CHVs) were trained on IFAS programme to enable them to provide IFAS health education together with the supplements to pregnant women in their homes. After training, just like the health care providers, the CHVs were provided with information, communication and education materials on IFAS for providing community based health education to pregnant women including: community health workers counselling guide and mothers’ leaflets, in both English and Swahili.

The third phase was follow-up phase where the pregnant women were followed-up until delivery of their babies culminating in collection of endline data. The control group were followed up by HCPs (specifically nurses) through the standard routine practice in health facilities which involved receiving IFAS health education and supplements at health facilities during antenatal care. The intervention group were followed up by CHVs on a weekly basis in their homes. During the weekly home visits, CHVs provided each pregnant woman with IFAS health education by counselling her on various IFAS topics including importance of taking IFAS tablets; causes, symptoms and effects of anaemia in pregnancy; dose, frequency and duration of IFA supplementation; common side effects and their management; how and when it is best to take IFAS; food sources of iron/folate; and enhancers/inhibitors of iron/folate absorption. After health education, they provided the entire week’s supply of IFAS tablets. Also, the CHVs encouraged pregnant women to attend antenatal care clinics to receive the other antenatal care services. A few weeks before delivery (from 36^th^ week of gestation), endline data was collected from the respondents who did participate up to the end of the study on socio-demographic characteristics, knowledge, source of IFAS information and levels of counselling on various IFAS topics, and attitude towards IFAS, in both control and intervention groups. The content of the questionnaire used for endline data collection was the same as that used for baseline data collection.

### 2.2 Study site, sampling and study population

Some of the details of methods adopted for recruitment of the pregnant women involved in this study as well as the ethical considerations have been published elsewhere in other articles [3, 22].

The study was conducted in Kiambu County in Kenya. Two stage sampling was adopted to identify one Sub-County (Lari) and five of its major public health facilities (Lari, Githirioini, Kagwe, Kagaa and Kinale) where the study was conducted. Sampling frame consisted of all Sub-Counties in Kiambu County. A criteria was used at each stage. The Sub-County with existing functional (active) community units formed the basis for the intervention, meaning its community health volunteers were actively involved in provision of community health services to community members. All the major public health facilities: with highest client/patient population turnover and with existing and functional (active) community units were used to implement the intervention. These were considered because of the low turn-over of antenatal clients in health facilities in Lari Sub-County.

Sample size expected to determine the effect of community based IFAS health education and distribution was calculated using the following formula for a binary outcome [23]:

$$n=(\frac{r+1}{r})\frac{\left(\bar{p}\right)\left(1-\bar{p}\right){\left(Z_{\beta }+Z_{\alpha /2}\right)}^{2}}{{\left(\text{D}\right)}^{2}}$$

D is the expected effect in IFAS health education of 20% (Control 25% to 45% intervention).

A consideration of 30% loss to follow-up was added to this sample, making a total sample size of 170. The final sample size per study group was 170 and in both groups was therefore **340**.

The study population consisted of all pregnant women who attended antenatal care in the selected health facilities who were: aged 18–49 years, below 33 weeks in their pregnancy gestation, not suffering from any chronic illness and who provided informed consent to participate in the study. Consecutive sampling, was used to include all accessible pregnant women as part of the sample. Consecutive sampling is considered the best type of non-probability sampling with best representation of entire population. All pregnant women who met the inclusion criteria were informed about the study and those who provided both verbal and written informed consent to participate in the study were recruited. Those residing in a community that had a functional community unit with active CHVS, who consented to have IFAS distributed to them in their homes together with health education by CHVs, formed the intervention group. Those residing in a community that did not have a functional community unit formed the control group, who received their IFAS health education and supplementation from fixed health facilities during antenatal care services, until the required sample size of 170 was reached.

### 2.3 Data collection tools and data collection process

A semi-structured interviewer-administered questionnaire consisting of 26 closed ended questions including; 11 on socio-demographic data, 9 on IFAS knowledge, 2 on sources of IFAS information, 4 on IFAS counselling content; and a likert scale with 19 statements on attitude and beliefs was developed, pre-tested and used for data collection in this study. To address any potential bias in data collection, training of four research assistants on research ethics and protocol and quality data collection was done at Kiambu level 5 hospital where the research questionnaires were pretested.

To ensure reliability of the questionnaire, a test re-test method was adopted in pre-testing, whereby a repeat pre-test was conducted after two weeks, and Cohen’s kappa statistic was used to measure the level of agreement of the results from the two pre-tests. The questions which were tested and re-tested included: on socio-demographic data: age, education level, occupation, income, gestation, parity and gravidity; on maternal knowledge: benefits of IFAS, frequency of taking IFAS, duration of taking IFAS, possible side effects of IFAS, how to manage the side effects, food sources that increase blood levels, consequences of not getting enough iron/folate, and signs and symptoms of anaemia; and the statements on attitude. All the questions repeated had a kappa value of above 0.7 after comparison thus the questionnaire was considered reliable, hence all the questions were retained. To ensure validity of the questionnaire, it was shared and discussed with experts from the Ministry of Health, division of nutrition, and the study supervisors. The feedback obtained from these experts and pre-testing results was used to refine the tool and improve its quality to ensure the questions were able to test what was intended.

The trained research assistants administered questionnaires to all pregnant women who met the inclusion criteria and consented to participate in the study at the health facilities selected for the study.

### 2.4 Determination of respondents’ level of knowledge

To assess the level of knowledge about IFAS during pregnancy, respondents were asked 9 questions: whether they have heard of IFAS or not, benefits of IFAS, frequency of use of IFAS, duration of taking IFAS, side effects, management of side-effects, effect of iron/folate deficiency, signs and symptoms of anaemia, and food sources for iron during pregnancy. A correct answer for each item was scored as “1” and incorrect answer scored as “0”. A summation of all the scores for each participant was done then converted into percent score. Based on references from other studies [24–26], those who scored above the average value (50%) were considered as somehow knowledgeable and those who scored below the average value were considered as less knowledgeable.

### 2.5 Determination of respondents’ attitude

The respondents’ attitude towards IFAS was assessed on 19 Likert scale items. A correct answer for each item was scored as “5” and incorrect answer scored as “1”. The scores were summed then converted into percent score. Using references from other studies [24, 25] and in consideration of majority score, a respondent was considered to have a positive attitude if they scored 70% and above and a negative attitude if they scored less than 70%. Women who reported not to have heard of IFAS were excluded in the assessment of attitude.

### 2.6 Data management and analysis

In order to examine effectiveness of the intervention, baseline and endline surveys were conducted in both study groups, using a similar questionnaire. To ensure adherence to optimal data quality standards, there was close supervision of the research assistants by the researcher.

Quantitative data at both baseline and endline was coded after collection then entered into the computer, cleaned and validated using Statistical Package for Social Sciences (SPSS) statistical software version 22. Data entry was done during the study to minimize errors. It was then exported to STATA version 14 for analysis. To ensure confidentiality, the computer access was restricted by password protection. Each questionnaire had a unique identifier to allow validation. Data cleaning and validation was done prior to analysis.

Descriptive statistics, including univariate analysis: simple proportions, n (%), for categorical variables and mean with standard deviation for continuous variables, were reported at baseline and endline. Characteristics of respondents were also described in both intervention and control groups. Knowledge, sources of IFAS information and whether counselling was offered on various IFAS topics to pregnant women were recorded. To ensure the change caused by the intervention was not by chance, baseline characteristics were similar in both groups. Homogeneity of study groups at baseline was determined by comparing socio-demographic characteristics of both groups. Bivariate analysis, using the chi-square test, was done for comparison between groups and multivariate analysis was used to control for confounders.

The analysis of effect of the intervention was done using a Difference-In-Difference (DID) regression model to compare outcomes between intervention and control groups before (baseline) and after (endline) the intervention. The changes in the dependent variables in the intervention group (from baseline to endline) were compared to changes in the control group (from baseline to endline) as shown in Table 1 below [27]. The intervention effect was measured by odds ratio and 95% confidence level of the interaction term between study groups (intervention and control) and period of survey (baseline and endline) in the multiple logistic regression model. A p-value of 0.05 was considered statistically significant. Since the same respondents who participated in the baseline are the same who participated in the end term evaluation, the analysis considered using a paired analysis with repeated measures instead of treating the respondents in baseline and endline as independents groups. Therefore, Generalized Estimation Equation (GEE) was applied in addition to allow correlation of these repeated observations over time since data are collected on the same participants across successive points in time. This is because GEE has been found to be more efficient for correlated longitudinal data, focusing on estimating the average response over the population ("population-averaged" effects) instead of estimating the effect of one or more covariates on a given individual [28–30].

**Table 1:** Intervention effect formula.

	Baseline		Endline
Intervention Group	Level of phenomenon before intervention (X)	Intervention introduced	Level of phenomenon after intervention (Y)
Control group	Level of phenomenon without intervention (A)		Level of phenomenon without intervention (Z)
	**Intervention Effect = (Y-X)–(Z-A)**		

Source: Kothari and Garg, 2014 pg 41.

### 2.7 Ethical considerations

Scientific and ethical approval was obtained from Kenyatta National hospital/University of Nairobi Ethics and Research Committee (KNH-ERC/A/90 protocol number–P706/11/2015) and research permit obtained from the National Commission for Science, Technology and Innovation (NACOSTI/P/18/81499/22319). Permission to conduct the study was obtained from managerial authorities at Kiambu County, Lari Sub-county and all health facilities involved.

Respondents were fully protected from any form of harm. Participation in the study was purely voluntary. The purpose of study was made clear to respondents who were required to give informed verbal and written consent. Emphasis on confidentiality and privacy were made clear at the time of consenting to participate and upheld throughout the study. No name appeared on the questionnaires so no participant identification with information could occur. Respondents were at liberty to discontinue from the study at any time without facing any adverse consequences. Information was kept confidential by restricted access and coding of questionnaires.

## Results

### 3.1 Socio-demographic characteristics of study respondents

A total of 340 pregnant women participated in the study during the baseline, of these 189 (56%) participated during the endline. The socio-demographic characteristics of respondents at baseline by study group are shown in Table 2. Most (n = 212, 62.4%) respondents were 20–29 years of age, with mean age of 25.6 (SD ± 5.6), married (n = 288, 84.7%), had a secondary level of education (n = 180, 53.4%), unemployed (n = 167, 49.1%), and earned less than USD 100 per month (n = 305, 93%). Whereas only 5.9% (n = 20) had attained tertiary level of education, among the 28.2% (96) that were in employment, only 3% (n = 10) were formally employed. In terms of gravidity, most (n = 223, 67.6%) of the women were multigravida. There was no statistical difference (p>0.05) in the characteristics of the pregnant women at baseline between the two comparison groups.

**Table 2:** Socio-demographic profile of the study respondents at baseline by group.

Variable	Total n (%) N = 340	Hospital n (%) N = 218	Community n (%) N = 122	Chi-square p-value
**Age of pregnant woman in years**				
Less than 20 years	43 (12.6)	27 (62.8)	16 (37.2)	0.899
20–29 years	212 (62.4)	138 (65.1)	74 (34.9)	
30 years and above Mean age (std)	85 (25) 25.6 (5.6)	53 (62.4) 25.6 (5.9)	32 (37.6) 25.7 (5.7)	
**Marital status**				
Married	288 (84.7)	183 (63.5)	105 (36.5)	0.32
Single	51 (15)	35 (68.6)	16 (31.4)	
**Education level**				
Primary	137 (40.7)	89 (65)	48 (35)	0.467
Secondary	180 (53.4)	111 (61.7)	69 (38.3)	
Tertiary	20 (5.9)	15 (75)	5 (25)	
**Occupation of pregnant woman**				
Unemployed	167 (49.1)	108 (64.7)	59 (35.3)	0.978
Casual employment	77 (22.6)	49 (63.6)	28 (36.4)	
Self-employed/Employed	96 (28.2)	61 (63.5)	35 (36.5)	
**Average income per month in USD**				
Less than100	305 (93)	194 (63.6)	111 (36.4)	0.274
100 and above	23 (7)	12 (52.2)	11 (47.8)	
**Parity**				
0	111 (33)	74 (66.7)	37 (33.3)	0.106
1	92 (27.4)	53 (57.6)	39 (42.4)	
2	80 (23.8)	50 (62.5)	30 (37.5)	
2 and above	53 (15.8)	41 (77.4)	12 (22.6)	
**Gravidity**				
Primigravida	107 (32.4)	74 (69.2)	33 (30.8)	0.171
Multigravida	223 (67.6)	137 (61.4)	86 (38.6)	
**Religion of pregnant woman**		
Protestant Christian	283 (83.5)	179 (63.3)	104 (36.7)	0.512
Catholic Christian	56 (16.5)	38 (67.9)	18 (32.1)	

### 3.2 Effect of community based approach on maternal knowledge towards IFAS

#### 3.2.1 Level of knowledge on IFAS among pregnant women

**Fig 2:**
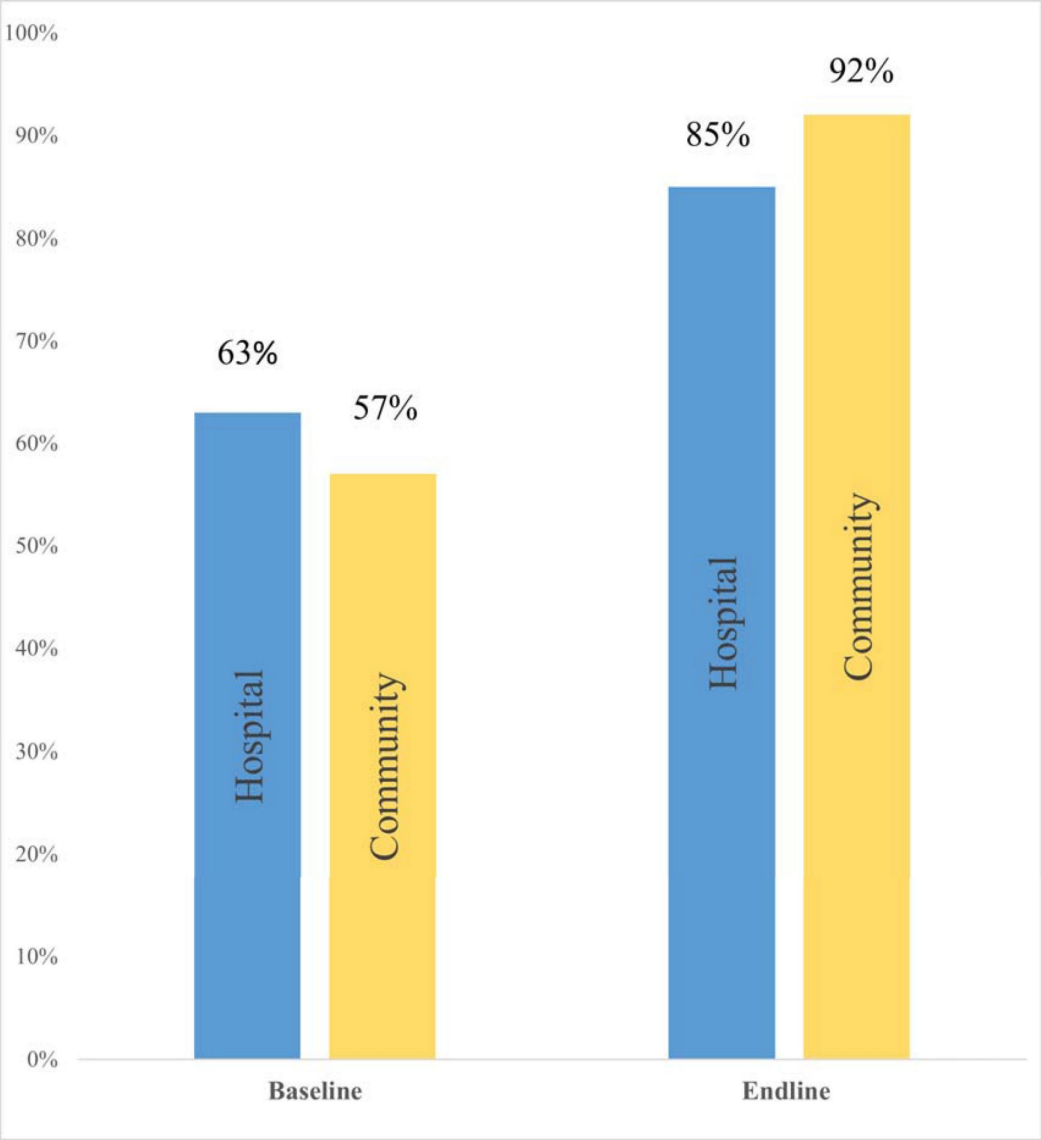
Maternal IFAS knowledge during study period. This refers to the level of maternal knowledge on IFAS at both baseline and endline for both the control (hospital) and intervention (community) groups.

Fig 2. shows a comparison between baseline and endline levels of maternal knowledge on IFAS for both hospital (control group) and community (intervention group) study groups. The improvement was 35 percentage points in the intervention group (from 57% to 92%) compared to 22 percentage points (from 63% to 85%) in the control group. The intervention had a net effect of 13 percentage points (35–22) improvement in IFAS knowledge level. However, it did not yield statistical difference (95%CI: -0.01, 0.27) since the Difference-In Difference (DID) between the two groups was 0.13.

#### 3.2.2 Factors associated with maternal knowledge on IFAS

Generalized Estimation Equation (GEE) results to assess the effect of the intervention and other potential factors on maternal IFAS knowledge are shown in Table 3. There was a highly significant (p<0.001) change in levels of IFAS knowledge between the two time points: the odds of being knowledgeable at endline was 3 times that at baseline (OR = 3.1:95%CI 1.7–5.8), adjusting for other soci-demographic factors. Employment status significantly influenced maternal knowledge, with casuals (p = 0.023) and the employed (p = 0.021) being more likely than the unemployed to be knowledgeable. However, the effect of the intervention did not yield statistical difference between the two groups on maternal knowledge on IFAS.

**Table 3:** Factors associated with maternal knowledge on IFAS.

Variable	Odds Ratio	P-value	[95% Confidence Interval]	
**Community vs. hospital**	0.776	0.291	0.486	1.242
**Endline vs. Baseline**	3.199	<0.001*	1.762	5.809
Interaction (group, time)**	2.545	0.077	0.903	7.171
**Age**				
20–29 years vs. <20 years	1.938	0.076	0.933	4.027
> 30 years vs. <20 years	1.626	0.297	0.652	4.050
**Level of education**				
Secondary vs. Primary	1.140	0.605	0.694	1.871
Tertiary vs. Primary	1.092	0.849	0.441	2.702
**Occupation**				
Casual vs. Unemployed	0.505	0.023*	0.280	0.911
Employed vs. Unemployed	0.574	0.021*	0.358	0.919
**Marital Status**				
Single vs. Married	1.076	0.836	0.539	2.146
Income in USD				
>100 vs. <100	1.536	0.377	0.593	3.979
**Parity**				
1 vs. 0	0.615	0.238	0.275	1.379
2 vs. 0	1.427	0.439	0.579	3.516
3 vs. 0	1.230	0.667	0.478	3.162
**Gravidity**				
Multigravida vs. Primigravida	1.147	0.720	0.542	2.428
**Religion**				
Catholic vs. Protestant	1.382	0.298	0.751	2.542

*Significant p-value at 0.05.

#### 3.2.3 Sources of IFAS information

**Fig 3:**
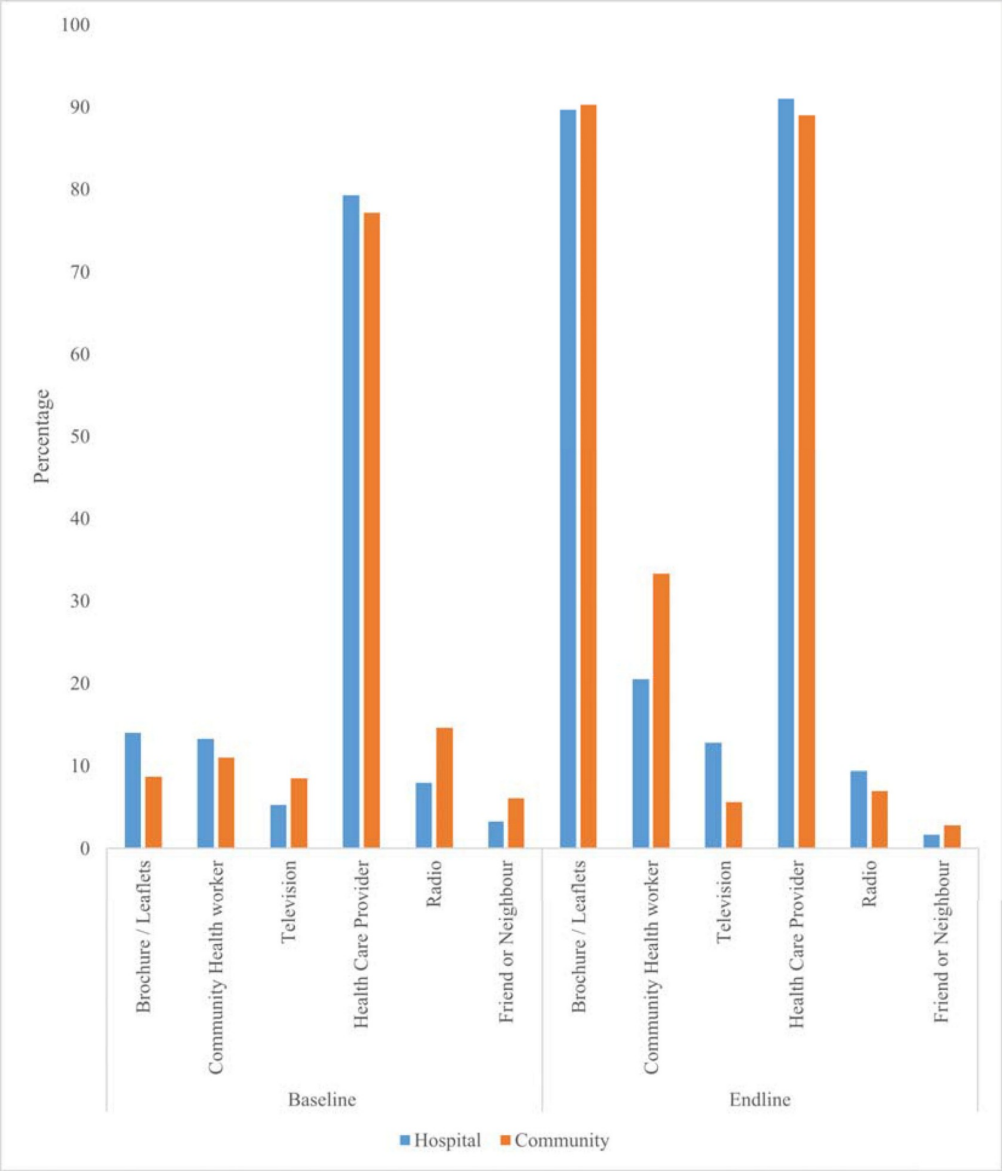
Sources of IFAS information at baseline and endline. This figure shows the sources of information on IFAS, where pregnant women reported to have received IFAS information from, at both baseline and endline for both the control (hospital) and intervention (community) groups.

During the study period, there was great improvement (from 67.3% at baseline to 99.6% at endline) in the proportion of respondents who had heard of IFAS. Fig 3. shows the sources of information in both control and intervention groups both before and after the intervention. In both groups, the proportion of respondents who obtained information from brochures/leaflets improved most during the study period. This was followed by those who obtained information from CHVs, with the improvement here being higher in the intervention group (11%-33.3%) as shown in Fig 3.

#### 3.2.4 Components of counselling provided as pregnant women received IFAS tablets in antenatal room

**Fig 4:**
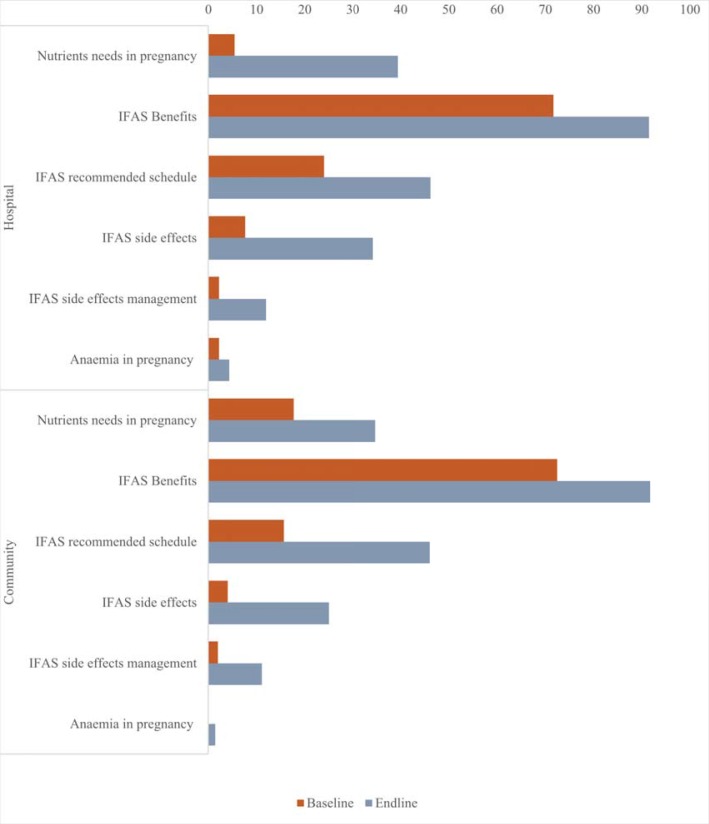
Advice provided during dispensing of IFAS in ANC room. This refers to the various aspects of IFAS counselling provided to pregnant women as they were issued with the IFAS tablets from the ANC room which is the usual routine practice.

During the study period, there was improvement in the proportion of pregnant women counselled on various components of IFAS as they received IFAS tablets from health care providers at the ANC room (where they are normally routinely dispensed from, in our local set-up), across study groups, as shown in Fig 4.

### 3.3 Effect of community based approach on maternal attitude towards IFAS

#### 3.3.1 Maternal attitude towards IFAS

**Fig 5:**
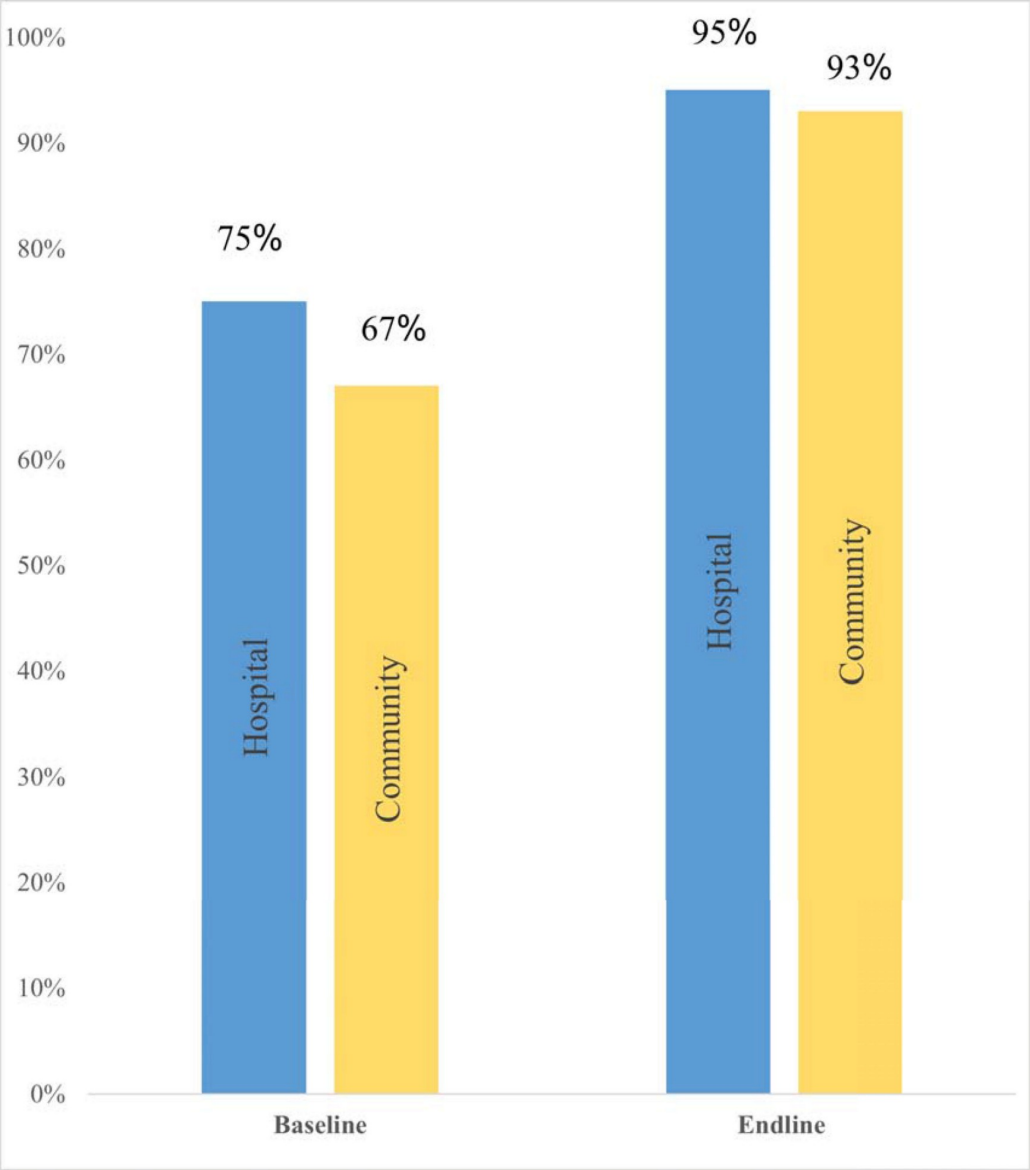
Maternal attitude towards IFAS during study period. This refers to the percentage score of maternal attitude at both baseline and endline for both the control (hospital) and intervention (community) groups.

Fig 5. shows comparison of maternal attitude towards IFAS between baseline and endline by study group. There was an increase in the proportion of pregnant women who had a positive attitude towards IFAS during the study period, across the study groups. The increase was higher at 26 percentage points (from 67% to 93%) in the intervention group compared to 20 percentage points (from 75% to 95%) in the control group. The intervention had a net effect of 6 percentage points (26–20) increase in positive attitude. However, it did not yield statistical difference (95%CI: -0.09, 0.20) since the DID between the two groups was 0.06.

#### 3.3.2 Factors associated with positive maternal attitude towards IFAS

Generalized Estimation Equation (GEE) results to assess the effect of the intervention and other potential factors on maternal attitude towards IFAS are shown in Table 4. There was a highly significant (p<0.001) change in proportion of those who had a positive attitude towards IFAS between the two time points: the odds of having a positive attitude towards IFAS at endline was 9 times that of baseline (OR = 9.2: 95%CI 3.0, 28.4), adjusting for other socio-demographic factors. However, the effect of the intervention did not yield statistical difference between the two groups on maternal attitude towards IFAS.

**Table 4:** Factors associated with positive maternal attitude towards IFAS.

Variable	Odds Ratio	P-value	[95% Confidence Interval]	
**Community vs. hospital**	0.769	0.428	0.402	1.471
**Endline vs. Baseline**	9.256	<0.001*	3.008	28.475
Interaction (group, time)**	0.710	0.658	0.156	3.234
**Age**				
20–29 years vs. <20 years	0.820	0.652	0.346	1.942
> 30 years vs. <20 years	0.943	0.913	0.328	2.709
**Level of education**				
Secondary vs. Primary	1.413	0.253	0.782	2.553
Tertiary vs. Primary	1.109	0.870	0.320	3.844
**Occupation**				
Casual vs. Unemployed	1.348	0.495	0.572	3.181
Employed vs. Unemployed	0.894	0.759	0.438	1.826
**Marital Status**				
Single vs. Married	0.997	0.996	0.400	2.490
Income in USD	1.151	0.805	0.378	3.506
>100 vs. <100	1.151	0.805	0.378	3.506
**Parity**				
1 vs. 0	2.101	0.288	0.534	8.265
2 vs. 0	2.710	0.189	0.612	12.008
3 vs. 0	1.846	0.447	0.380	8.968
**Gravidity**				
Multigravida vs. Primigravida	0.452	0.266	0.111	1.833
**Religion**				
Catholic vs. Protestant	0.701	0.328	0.344	1.429

*Significant p-value at 0.05;

**DID.

## Discussion

The aim of this study was to determine the effect of using Community Health Volunteers (CHVs) to provide IFAS health education to pregnant women on knowledge, attitude, quality of counselling and sources of information on IFAS. Key findings were that community based health education improved (i) maternal knowledge on IFAS (ii) general awareness on IFAS (iii) the proportion of women counselled on various IFAS aspects at ANC, and (iv) positive attitude towards IFAS among pregnant women.

At the end of the study, nearly all the pregnant women had heard of IFAS compared to about a third of them who had not heard of IFAS at the beginning of the study. In addition, there was a greater improvement in the proportion of pregnant women who scored high for levels of IFAS knowledge, especially in the intervention group. This shows that more pregnant women were reached and counselled on IFAS by CHVs compared to those reached routinely through health facilities. Previous studies have shown that community distribution of IFAS reach a higher population of women and attain more positive behavior change, including knowledge, compared to antenatal (ANC) provision alone [31]. Iron and folic acid supplementation has been shown to be a cost-effective and common strategy used to control iron deficiency anaemia [32, 33]. Since maternal knowledge has been shown to affect the uptake of IFAS, improving knowledge and awareness lead to better IFAS uptake [15]. Existense of community health structures with CHVs playing a key role is an opportunity to improve maternal knowledge on IFAS and hence increase uptake of this high impact intervention.

Though most pregnant women were aware of IFAS, the proportion of of those who scored high on levels of IFAS knowledge was low. This shows the need to constantly train CHVs and health care providers in order to improve the quality of health education. Low quality of health education is probably due to inadequate dissemination of existing IFAS guidelines. To increase uptake, women need to know what is offered to them. A similar study in Saudi found that although 88% of the women had heard of folic acid, only 53.8% of them had accurate information about its benefits [34].

Counselling offered at health facilities was found to be inadequate similar to what was found by Nisar and Titaley [35, 36] in their studies. Grebremedhin and colleagues [15] pointed to the need for constantly training and updating health care providers for them to provide quality and effective counselling on IFAS. Training provided during this study was the first among nurses in Kiambu County pointing to irregular and often inadequate capacity building for health care providers. The knowledge and skills gained contributed to the high knowledge scores among pregnant women at endline data. Beyond the Health Care Providers (HCPs), there is need to explore other strategies for creating awareness and educating the pregnant women on IFAS that can compliment ANC provision. This will most probably increase maternal IFAS knowledge and consequently its utilization. The CHVs provide such an opportunity since they are also able to closely follow-up pregnant women and provide health education at the community level, as evidenced in this and other studies [37].

Using CHVs to provide IFAS health education was successful in improving IFAS knowledge among pregnant women. Pregnant women who were employed were more likely to be knowledgeable than the unemployed women. These women had also attained a higher levelof education. Formal education influences level of income and creates opportunities for gathering information and knowledge on IFAS as shown in studies in other countries [38, 39]. Clients with high income are more likely to have attained high education level and hold formal employment, or own good businesses [6, 39–42]. These findings are similar to a recent study in Ethiopia where there was a positive association between educational status, family income and nutritional knowledge of pregnant women [43]. This calls for more opportunities to empower women.

It is noteworthy that, the source of IFAS information reported by pregnant women that resulted in the greatest improvement between baseline and endline in this study was brochures, followed by CHVs, then HCPs. Possible explanations for these findings may include; (a) a lot of information, education and communication materials were distributed during trainings including posters, brochures, mothers IFAS calendars and counselling guides that were used for counselling the pregnant women (b) Pregnant women were provided with brochures/leaflets to take home for reference (c) community health volunteers are closer to the community leading to better interpersonal communication skills than health care providers (d) health care providers may still be overworked as earlier studies noted and may therefore not have adequate time for counselling pregnant women [14, 44] (e) all pregnant women were required to come for antenatal care and HCPs had an obvious opportunity to counsel them.

Increase in proportion of those who reported CHVs as their source of IFAS information at endline indicates that follow-up by CHVs to pregnant women was successful. Formal involvement of CHVs in education and distribution of IFAS can significantly improve both education and uptake by closely following up the pregnant women at the community level [45, 46]. Though HCPs have traditionally been the main source of health information for clients and patients [47], CHVs have emerged as an important source of health information as well [11, 14, 48]. As Titaley stated, strengthening counselling sessions during antenatal care alone without improving community-based education is not sufficient to improve IFAS uptake [35]. Since, CHVs are an important link between health services and community, their effectiveness is highly dependent on proper training, provision with information, education and communication materials and supportive supervision. If properly trained and facilitated, CHVs could play a vital role in increasing uptake of health interventions like IFAS as well as acting as a credible source of health education as reported by Saprii and colleagues [49]. Hence, the need to properly train CHVs and formalize their operations in relation to IFAS services.

There was an increase in the proportion of pregnant women counselled on various components of IFAS during ANC visits as well as increased awareness. Increased IFAS awareness and health education coupled with quality counselling and effective communication has been shown to improve IFAS uptake [15, 16, 50]. This necessitates the need for targeted and focused information and training on importance of IFAS during pregnancy [18]. This further indicates the need to expand strategies aimed at creating awareness on IFAS both at health facility level and community level so as to further facilitate health education of clients. Currently, the CHVs are not allowed to distribute IFAS supplements with education through home visits in the country. Using CHVs to do this has proved to be of great benefit in this study and would be a promising strategy if adapted formally.

The attitude and perception of pregnant women is based on the information they have which in turn determines their practices [46]. A study conducted in Ethiopia showed a positive association between maternal attitude and nutritional knowledge of pregnant women [43]. Since most clients trust their health worker [36], investing in giving women adequate information, will most probably lead to a positive attitude which will in turn improve IFAS uptake and practices. This study aimed at improving maternal IFAS knowledge and attitude and thus consequently uptake of IFAS. The aim was to change individual behaviour by reaching pregnant women in their local community settings as practiced by Bruce and colleagues [51] using the strategy of education/behaviour change [52]. During this study, attitude towards IFAS improved among pregnant women especially those in the intervention group. The odds of having a positive attitude towards IFAS at endline was 9 compared to the odds at baseline. There was an overall positive change in beliefs, opinions and perception of pregnant women towards IFAS during this study. These findings are similar to a nutrition education intervention study done among postpartum women that demonstrated significantly greater improvement in overall positive health beliefs and practices that helped women to give up their negative beliefs, perceptions and practices in relation to IFAS [53]. Elsewhere, maternal attitudes have been associated with less frequent use of various supplements [54]. Individualized counselling of pregnant women can address concerns and negative attitudes through discussions and clarification. This is an iterative process that CHVs are well suited to carry out, as demonstrated in this study.

This study had several limitations: (i) The participants were only followed during their pregnancy period, giving a limited time for follow-up. (ii) High loss to follow-up. Though impact of loss to follow up was minimized by increasing the sample size by the estimated rate of loss to follow-up of 30%, this was increased by other unavoidable circumstances. A prolonged industrial action by government employed health workers during the study period led to higher than the anticipated loss to follow up due to disruption of services. (iii) The intervention was community based and without randomizatiom. The effect of this limitation was minimized by use of a control group. This avoided extraneous variation resulting both from passage of time and from non-comparability of the test and control areas. In addition, due to the low turn-out of antenatal clients in the Sub-County, consecutive sampling was used to include all accessible pregnant women as part of the sample. (iv). Both the HCPs and CHVs were trained instead of CHVs alone, potentially introducing confounding of the effect. This could not be avoided because CHVs needed to refer clients for other antenatal care services and were being supervised and issued with IFAS tablets by the nurses, who therefore needed to be trained as well. (v) The study results were prone to recall bias and subjectivity because the study greatly relied on verbal reports from the interviewees. This challenge was mitigated by training of interviewers as well as double questioning to identify any inconsistencies in the interview responses. (v) Generalizations of the study findings to other areas with different socio-demographic characteristics may be difficult since the study was limited to one Sub-County.

## Conclusion

Implementation of a community based approach of IFAS health education improved the maternal knowledge and positive attitude towards IFAS. In addition, the approach led to increase in proportion of women receiving IFAS brochures and that were counselled on various IFAS aspects. Follow-up of pregnant women by CHVs played a great role in these results. Existing community health structures in Kenya with CHVs providing a link between community and health facility is a viable opportunity for providing health education and distributing IFAS in order to improve its uptake. For the benefits of this approach to be realized, CHVs need to be trained and regularly supervised, in addition to amending the policy to allow them to distribute IFAS supplements together with health education to pregnant women in their homes.
